# Genetic Dissection of New Genotypes of Drumstick Tree (*Moringa oleifera* Lam.) Using Random Amplified Polymorphic DNA Marker

**DOI:** 10.1155/2013/604598

**Published:** 2013-05-21

**Authors:** Shamsuddeen Rufai, M. M. Hanafi, M. Y. Rafii, S. Ahmad, I. W. Arolu, Jannatul Ferdous

**Affiliations:** ^1^Food Crops Laboratory, Institute of Tropical Agriculture, Universiti Putra Malaysia, 43400 Serdang, Selangor, Malaysia; ^2^Department of Crop Science, Faculty of Agriculture, Universiti Putra Malaysia, 43400 Serdang, Selangor, Malaysia

## Abstract

The knowledge of genetic diversity of tree crop is very important for breeding and improvement program for the purpose of improving the yield and quality of its produce. Genetic diversity study and analysis of genetic relationship among 20 *Moringa oleifera* were carried out with the aid of twelve primers from, random amplified polymorphic DNA marker. The seeds of twenty *M. oleifera* genotypes from various origins were collected and germinated and raised in nursery before transplanting to the field at University Agricultural Park (TPU). Genetic diversity parameter, such as Shannon's information index and expected heterozygosity, revealed the presence of high genetic divergence with value of 1.80 and 0.13 for Malaysian population and 0.30 and 0.19 for the international population, respectively. Mean of Nei's gene diversity index for the two populations was estimated to be 0.20. In addition, a dendrogram constructed, using UPGMA cluster analysis based on Nei's genetic distance, grouped the twenty *M. oleifera* into five distinct clusters. The study revealed a great extent of variation which is essential for successful breeding and improvement program. From this study, *M. oleifera* genotypes of wide genetic origin, such as T-01, T-06, M-01, and M-02, are recommended to be used as parent in future breeding program.

## 1. Introduction

Drumstick tree (*Moringa oleifera* Lam.), a short to medium height tree with luxurious evergreen leaves, was said to have originated from Himalayan tract in northwestern part of India [1–4]. The tree has a true diploid chromosome 2*n* = 28 with a distinguished tripinnate leaves having yellow or white petiole streaks [5, 6]. Moringa is potentially one of the planet's most valuable plants, at least in humanitarian terms [7] and has been regarded as a wonder tree due to its great economic importance and uses [3, 7]. Its pods were reported to have a protein content ranging from 20 to 30%, with a high vitamin C content. The moringa seeds were found to exhibit the property of natural coagulants/flocculants, which allows for growing of the tree for the purpose of usage by water and sewage treatment plant to clear turbidity in drinking water and sludge in sewage [8]. Similarly, the nutritive value of this plant for animals has been documented by Mendieta-Araica et al. [1], who reported that moringa contains large amount of crude protein, iron, zinc, and high concentration of vitamins A, B, and C in its foliage sample which makes it a very good feed and fodder for animals to browse and graze upon [9].

With respect to oil quality, *M. oleifera* seed concentrate contains about 35–45% seed oil, having odourless and colourless physical properties [10]. The edible oil is highly nutritious and is extracted by boiling the seeds with water and collecting the oil from the surface of the water [9, 11]. The seed oil has high concentration of oleic acid (>73%) coupled with low polyunsaturated fatty acid, which gives the oil an outstanding and remarkable oxidative stability properties. The suitability of *M. oleifera* seed oil as biodiesel feed source has been tested and recommended by Da Silva et al. [12], who reported that the oil could be used as pure biodiesel or petrodiesel mixture on engine after converting it to fatty acid methyl esters (FAME) through the process of transesterification in the presence of sodium hydroxide (NaOH) as catalyst.

Moreover, despite the great economic importance of this plant in terms of nutritional, social, and environmental benefits, the genetic diversity pattern, genetic makeup, and agronomical requirement needed for successful breeding and improvement, domestication, and large scale cultivation are yet to be established. This obstacle is an impediment to a successful production and commercialization of moringa and its related products [6]. Also, the knowledge of genetic diversity of tree crop is very important for rational planning of conventional, modern breeding, and improvement program for the purpose of improving the yield and quality of its produce [9, 13].

In other words, the use of molecular markers, such as inter-simple sequence repeat (ISSR), random amplified polymorphic DNA (RAPD), and simple sequence repeat (SSR), has gained popularity as a genetic diversity assessment methods of tree and oil seed crops [14–16]. Molecular methods of genetic diversity study are a fast, efficient, reliable, and simple means of establishing genetic diversity pattern in plant [17]. The RAPD as one of the numerous molecular markers has been reported to be a reliable, reproducible, cost effective, fast, and less tedious marker, which is widely used in the field of plant breeding and molecular genetics due to its outstanding quality [18].

Therefore, this research work will study the genetic diversity of twenty new genotypes of *Moringa oleifera *from two populations with the aim of studying genetic diversity pattern in relation to their geographical origin, and dissection of germplasms as a means of initiating the breeding programme in the nearest future.

## 2. Materials and Methods

### 2.1. Plant Materials

These were made up of seeds of twenty new genotypes of *M. oleifera *collected from six different countries (Table 1). The moringa genotypes prior to their collection were found in the wild growing in their natural form. The countries of origin are Virgin Island, USA, Thailand, India, Tanzania, Taiwan, and Malaysia. The collection was principally made by the Asia Vegetable Research and Development Center (AVRDC) or World Vegetable Centre, Taiwan (15 accessions classified as international population), and the Institute of Tropical Agriculture, universiti Putra Malaysia (5 accessions classified as Malaysian population). The collected seeds were germinated and raised in nursery, Universiti Putra Malaysia Agricultural Park (TPU) for two months, exposed to the hardening process in the last ten days of nursery, then transplanted out to the University's agricultural experimental farm in Puchong (02°N59.035′, 101°E38.913′), Selangor, Malaysia. Young and disease-free leaves of *M. oleifera *were collected for each of the genotypes during the early hours of the day; the leaves sample were wrapped in aluminum foil and labeled and kept in the freezer at −10°C.

### 2.2. DNA Extraction Protocol

Six hundred *μ*L of extraction buffer (100 mM of Tris-HCl, 20 mM of EDTA, 1.4 M NaCl, and 5% SDS) was added to 10 mg leaf sample and ground with mortar and pestle without liquid nitrogen according to Ferdous et al. [19]. The finely ground leaf tissue was transferred into 2 mL centrifuge tube. Four hundred *μ*L of 2X CTAB solution, 100 mM of Tris-HCl, 20 mM of ethylenediaminetetraacetic acid di-sodium salt (EDTA), 1.4 M of sodium chloride (NaCl), 2% (w/v) CTAB, and 1% (w/v) of polyvinyl pyrrolidone (PVP) and 400 *μ*L chloroform: isoamyl alcohol : phenol (24 : 1 : 5%) mixture were then added to the leaf tissue containing the extraction buffer. After mixing through vortex and centrifuge, the supernatant was transferred to another tube. Two-third of volume of isopropanol was added and incubated at room temperature for 10 to 15 min. Centrifuged supernatant was then removed and the DNA pellet was washed using 70% ethanol; afterwards the DNA pellet was air-dried and dissolved into 50 *μ*L TE buffer.

### 2.3. DNA Quantification and Dilution

The quantification of DNA was carried out using NanoDrop ND-1000 spectrophotometer (NanoDrop Technologies, Wilmington, USA). The DNA was again requantified by running it through 1% agarose gel electrophoresis with 1 × TAE buffer for 30 min and viewed under UV light after staining it with Midori green DNA stain (Nippon Genetics Inc., Germany). The dilution was done with sterile distilled water to ensure that all of the DNA samples have equal concentration of 100 ng/*μ*L.

### 2.4. RAPD Polymerase Chain Reaction Procedure

According to the company instruction (Promega), 5 *μ*L of 5X Green GoTaq Flexi Buffer, 3 *μ*L MgCl_2_ solution (25 mM), 0.5 *μ*L PCR nucleotide mix (10 mM each), 0.2 *μ*L primers (0.4 *μ*mol), and 1.0 U of *Taq *DNA polymerase were used for 25 *μ*L of PCR reaction including 1 *μ*L DNA template directly used after extraction [19]. In RAPD analysis, the following condition was used: initial denaturation at 94°C for 1 min followed by 45 cycles of denaturation done at 94°C for 1 min, annealing was done at 34°C for 1.5 min, and extension was done at 72°C for 2 min and a final extension at 72°C for 5 min [20]. The amplified PCR products were subjected to electrophoresis on 3% (w/v) MetaPhor agarose gel at 75 volt for 70 minutes. The gel was stained with ethidium bromide and visualized under ultraviolet (UV) light.

### 2.5. Band Scoring

The image of the gel acquired in JPEG format was imported into UVIdoc 99.02 for band scoring. The band sizes were estimated based on DNA ladder (Promega Inc.). The absence and presence of band were scored in a binary model of 0 and 1, respectively. Band scoring was carried out only on those bands that are clear and reproducible and then those that are >50 bp. The data obtained at the end of the scoring was transferred and saved in Microsoft excel sheet.

### 2.6. Data Analysis

Data of the twelve primers were analyzed to obtain the information on genetic diversity of the 20 moringa accessions (Table 2). Genetic similarity among the genotypes and principal component analysis (PCA) were calculated using NTSYS-pc 2.1. Cluster analysis was also carried out using the unweighted pair-group method with arithmetic average (UPGMA) based on the Nei's genetic distance matrix and dendrogram was drawn to show the clustering pattern of the different genotypes using NTSYS-pc. The percentage polymorphism of the bands (PPB), effective alleles (ne), genetic diversity index (h), Shannon's information index (I), and Nei's gene diversity were calculated using POPGEN 1.31 software. Analysis of molecular variance was conducted using GeneAIEx 6.5 to partition the variation present in the germplasm and at the same time test the variance component for RAPD phenotype. 

## 3. Results

### 3.1. Screening of Primers

**Figure 1:**
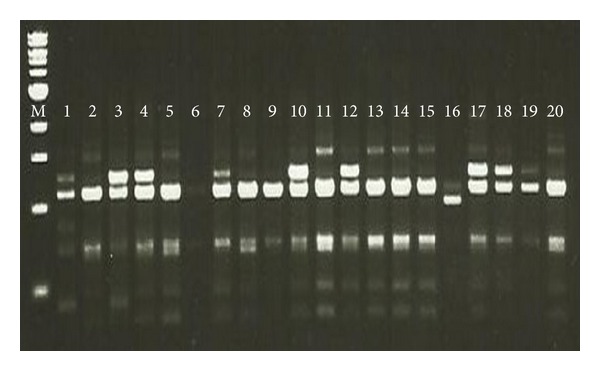
Gel picture of twenty genotypes of *Moringa oleifera* RAPD profile.

A total of 24 RAPD primers were used to study the genetic diversity of twenty genotypes of *M. oleifera* (Figure 1). Out of these primers, only 12 showed as distinct, reproducible polymorphic bands. A total of 108 polymorphic fragments were generated by these 12 primers with an average of 9.0.

*Genetic Diversity within the Two Populations. *The mean percentage polymorphic loci in the two populations (international and Malaysian) were calculated to be 75.73 and 32.70, respectively (Table 3). The observed number of alleles in the two populations from Taiwan and Malaysia is 1.50 and 0.71, respectively with 1.26 as the mean value for effective alleles for the two countries. With the Hardy Weinberg equilibrium assumption in place, Shannon's information index and expected heterozygosity for Taiwan population were 0.3 and 0.19 and those of Malaysian population were 0.18 and 0.13, respectively, while mean Nei's gene diversity for the two populations were estimated to be 0.20.

Furthermore, in order know the source of genetic variation for these Moringa genotypes, RAPD profile was analyzed using AMOVA. This was aimed to partition all the sources of variation existing in the germplasm into two major groups. The result revealed that 95% of the total genetic variation occurred as a result of variation within the population, while variation among the populations accounted for the remaining 5% of the total genetic variance (Table 4). Also the genetic variance among the population as indicated by the result (*F*_st_ = 0.16) was significant at 5% probability level when permutation test was conducted.

### 3.2. Cluster Analysis

**Figure 2:**
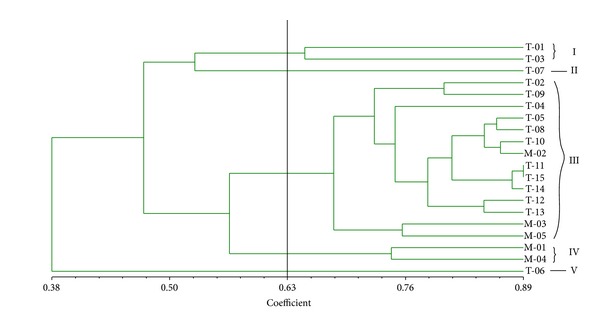
A dendrogram display of twenty accessions of *Moringa oleifera. *

Cluster analysis based on Jaccard's genetic similarity coefficient showed high level of genetic variation among the genotypes from the two countries. The similarity coefficient ranged from 0.38 to 0.89, with T-11 and T-15 genotypes found to have highest genetic similarity (0.89), while T-06 together with T-07 possessed least similarity coefficient (Figure 2).

In addition, a dendrogram was constructed using UPGMA cluster analysis to show the genetic relationship among the twenty genotypes from different geographical backgrounds. From this dendrogram, the twenty genotypes were grouped into five major clusters at a coefficient of 0.63. Cluster III, with highest number of members, had 14 genotypes, followed by cluster I (T-01 and T-03) and cluster IV (M-01 and M-04) with two genotypes each. Clusters II (T-07) and V (T-06) have one genotype each and were ranked the least populated clusters (Table 5). 

### 3.3. Principal Component Analysis

**Figure 3:**
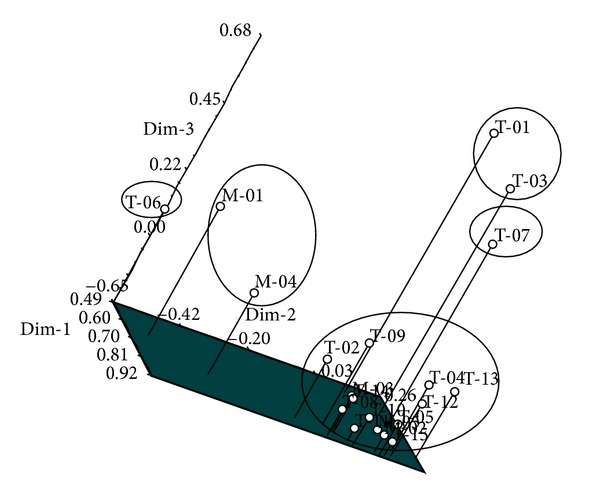
Three-dimensional principal components analysis of 20 accessions of *Moringa oleifera. *

The principal component analysis (PCA) carried out with RAPD *M. oleifera* profile classified the twenty genotypes of *Moringa* into five major groups with two genotypes from Malaysia occupying one group. This grouping pattern corresponds with that of clustering analysis as shown by the dendrogram. Dimension one of the PCA ranges from 0.49 to 0.92, while dimension two ranged from −0.65 to 0.26 and dimension three ranged from −0.65 to 0.68 (Figure 3).

## 4. Discussion

Effective and efficient genotyping of any plant species through RAPD requires a careful selection of suitable primer combination in order to get detail and informative result. High level of genetic polymorphism detected by these markers is in agreement with the assumption that outcrossing plant species from natural population will have higher level of genetic diversity when compared to in-breeding plant species. This finding agrees with earlier report on similar out-crossing plant species, such as Jatropha [23] and other oil plant species. High value of Shannon's information index (0.295) for international population as compared to Malaysian population (0.184) suggests that members of this population are more diverse. This is also obvious from the way the accessions cluster together.

Additionally, high level of genetic differentiation in these two populations as reflected by the genetic diversity parameters, such as Shannon's information index, expected heterozygosity, percentage polymorphism, and others, are pointing to the fact that there is wild variability in these populations of *Moringa* and this is very important for successful crossing and improvement programs in future. This observation follows a similar trend with the result of genetic diversity study on 75 accessions from Sudan and Guinea savanna zones of Nigeria, where six polymorphic primers of RAPD origin gave a total of 42 polymorphic bands [9].

Furthermore, interaction between various ecological and biological factors, such as genetic drift, gene flow, selection, and mating system, affects the genetic structure of any plant populations [24]. The overall genetic variability and differentiation pattern observed in these *M. oleifera *populations are in agreement with those of other outcrossing plant species [14, 25, 26]. As shown by these results, the two populations of *M. oleifera* exhibit moderate *F*_st_ value in order to demonstrate low significant genetic differentiation among the populations. However, higher genetic differentiation and diversity were observed within the populations of *M. oleifera *and this indicates a relatively restricted variability as expected. This pattern of population structure has been previously reported in other out crossing plant species [27, 28].

Moreover, clustering analysis showing wide range of similarity coefficient showed that, there is high level of genetic variation within the two populations. The *M. oleifera* genotypes from the two populations were seen clustering together in the same group. This shows that there is no any distinct relationship between the geographical origin and the genetic distance as shown in the dendrogram. This finding implies that the genetic divergence within and between these two populations could not be explained by their geographical distance. This finding also means that isolation as a result of distance cannot be said to have been responsible for the divergence observed in these population [24].

In conclusion, these findings have proven that genetic divergence is very high in these populations and it can therefore be inferred from the data that the *Moringa* populations will be a very good germplasm material for the future breeding and improvement of this economically important tree crop. Genotypes that are far apart based on their genetic similarity coefficient (like T-01, T-06, M-01, and M-02) should be selected for future breeding.

## Figures and Tables

**Table 1:** List of moringa genotypes and their countries of origin.

Serial number	Genotypes ID	Pedigree/cultivar name	Origin country
1	T01	Virgin Islands Drum Stick	USA
2	T02	Ma Rum01	Thailand
3	T03	Ma Rum02	Thailand
4	T04	Ma Rum03	Thailand
5	T05	Ma Rum04	Thailand
6	T06	Ma Rum05	Thailand
7	T07	Ma Rum Khaw Nheaw	Thailand
8	T08	Ma Rum06	Thailand
9	T09	Ma Rum07	Thailand
10	T10	Ma Rum K	Thailand
11	T11	Tnau-1	India
12	T12	Rca Moringa	Tanzania
13	T13	Ma Rum C	Thailand
14	T14	Drumstick Tree Pkm-1	India
15	T15	La-Mu W	Taiwan
16	M01	ITA-UPM01	Malaysia
17	M02	ITA-UPM02	Malaysia
18	M03	ITA-UPM03	Malaysia
19	M04	ITA-UPM04	Malaysia
20	M05	ITA-UPM05	Malaysia

**Table 2:** RAPD polymorphic primers and their sequence ID.

No.	Synthesis ID	Sequence	*μ*g	nmol
1	OPA_17	5′-GAC CGC TTG T-3′	101	33.5
2	OPA_19	5′-CAA ACG TCG G-3′	93	34.5
3	OPB_17	5′-AGG GAA CGA G-3′	85	27.1
4	OPBC_10	5′-AAC GTC GAG G-3′	160	52
5	OPBD_18	5′-ACG CAC ACT C-3′	179	60.6
6	OPBD_19	5′-GGT TCC TCT C-3′	181	61.1
7	OPF_20	5′-GAG GAT CCC T-3′	95	31.3
8	OPH_19	5′-CTG ACC AGC C-3′	100	33.7
9	OPO_3	5′-CTG TTG CTA C-3′	103	34.6
10	OPM_6	5′-CTG GGC AAC T-3′	100	32.9
11	OPM_8	5′-TCT GTT CCC C-3′	110	37.5
12	OPQ_2	5′-TCT GTC GGT C-3′	104	34.5

**Table 3:** Genetic diversity in *M. oleifera* germplasm as revealed by RAPD.

Population		*N*	Na	Ne	*I*	He	P (%)
International		15.000	1.500	1.308	0.295	0.188	72.73
Malaysia		5.000	0.709	1.220	0.184	0.125	32.73
Grand total	Mean	10.000	1.105	1.264	0.239	0.156	52.73
	SE	0.338	0.065	0.024	0.018	0.013	20.00

*N*: number of genotypes per each populations.

Na: observed number of alleles.

Ne: effective number of alleles Kimura and Crow [21].

*I*: Shannon's information index Lewontin [22].

He: expected heterozygosity.

P (%): Percentage of polymorphism.

**Table 4:** Genetic divergence differentiation with analysis of molecular variance.

Source	Degree of freedom	Sum square	Mean square	Estimated variance	variation (%)	*F*_st_	*P* value
Among population	1	15.733	15.733	0.558	5	0.160	<0.001
Within population	18	207.867	11.548	11.548	95		<0.001

Total	19	20	233.600	12.106	100		

**Table 5:** Name of clusters and their corresponding genotypes.

Cluster I	T-01, T-03
Cluster II	T-07
Cluster III	T-02, T-04, T-05, T-08, T-09, T-10, T-11, T-12, T-13, T-14, T-15, M-2, M-03, M-04
Cluster IV	M-01, M-04
Cluster V	T-06
